# *armA* and Aminoglycoside Resistance in *Escherichia coli*

**DOI:** 10.3201/eid1106.040553

**Published:** 2005-06

**Authors:** Bruno González-Zorn, Tirushet Teshager, María Casas, María C. Porrero, Miguel A. Moreno, Patrice Courvalin, Lucas Domínguez

**Affiliations:** * Universidad Complutense de Madrid, Madrid, Spain;; † Laboratorios Ovis, Barcelona, Spain;; ‡ Institut Pasteur, Paris, France

**Keywords:** escherichia coli, animal isolate, armA, 16S rRNA methylase, aminoglycoside resistance, IncN, transposition, veterinary monitoring

## Abstract

We report *armA* in an *Escherichia coli* pig isolate from Spain. The resistance gene was borne by self-transferable IncN plasmid pMUR050. Molecular analysis of the plasmid and of the *armA* locus confirmed the spread of this resistance determinant.

Aminoglycosides are used to treat a broad range of life-threatening infections in humans and animals (1,2). Thus, surveillance of resistance to these antimicrobial agents and study of the corresponding mechanisms in pathogenic bacteria remain ongoing challenges in microbial research (3). Since 1996, the Spanish Veterinary Antimicrobial Resistance Surveillance Network has monitored antimicrobial resistance in both healthy and ill animals, with the aim of assessing the state of veterinary antimicrobial resistance in Spain (4–6). *Escherichia coli* is used as a model bacterium for the analysis of isolates from sick animals. Veterinary diagnostic laboratories from official and private sectors provide the isolates, following a classic passive monitoring system with a centralized analytic approach. To avoid bias, a single isolate per farm per year (or animal in the case of pets) is studied. The identity of the farm (or owner) is kept confidential to facilitate participation. In addition, isolates are included only if their antimicrobial susceptibility has not been determined before being sent to the network. Although formal sampling is not performed, involvement of several laboratories throughout the country elicits an accurate estimate of antimicrobial susceptibility in Spain (4–6). From 1996 to 2003, the number of isolates tested was >2,300 for *E. coli* and 700 for *Staphylococcus aureus*.

## The Study

*E. coli* MUR050 from the feces of a diarrheic pig was isolated in 2002. Routine antimicrobial susceptibility testing showed unusual resistance to amikacin. The isolate was also resistant to other aminoglycosides (streptomycin, gentamicin, and neomycin, but not apramycin), as well as to members of other classes (sulfonamides, trimethoprim, quinolones). Because of this unusual phenotype, the standard identification procedure (Gram staining, oxidase, indol, methyl red, citrate, and Vogues-Proskauer) (6) was confirmed by partial sequencing of the ribosomal 16S rRNA gene (data not shown) (7). Additional study of antimicrobial susceptibility by Etest (AB Biodisk, Solna, Sweden) indicated that the strain was also highly resistant to the 4,6-disubstituted deoxystreptamines netilmicin and tobramycin (Table). We initiated an investigation to find the molecular determinants of this pattern. Plasmid DNA purified from MUR050 (Qiagen, Inc., Chatworth, California, USA) was used to transform *E. coli* INVαF´ (Invitrogen, Paisley, United Kingdom) (8) to yield strain INVαF´ (pMUR050), which was highly resistant to aminoglycosides and sulfonamides. Resistance to aminoglycosides and the sulfonamides could be transferred from MUR050 to other *E. coli* by conjugation at a frequency of ≈1 × 10^–4^ per donor colony-forming unit (9).

**Table:** Minimum inhibitory concentrations (MICs) of various antimicrobial agents for *Escherichia coli* MUR050 and *E. coli* with and without *armA*

	MIC (μg/mL)*				
*E. coli*	Amikacin	Tobramycin	Netilmicin	Gentamicin	Ciprofloxacin
MUR050	>256	>256	>256	>256	>32
INVαF´	0.5	0.5	0.5	0.75	0.002
INVαF´ (pMUR050)	>256	>256	>256	>256	0.002
INVαF´ (p18MUR050)	>256	>256	>256	>256	0.002
INVαF´ (p*armA*)	>256	>256	>256	>256	0.002

*MICs were determined by Etest (AB Biodisk).

Search for antimicrobial resistance determinants by polymerase chain reaction (PCR) using plasmid pMUR050 DNA as template indicated the presence of genes *sul1* for resistance to sulfonamides, *ant3*´´9 for resistance to streptomycin-spectinomycin, and *aph3*´-I for resistance to kanamycin and neomycin. Plasmid pMUR050 DNA was digested with PstI and ligated with pUC18 DNA; the ligation mixture was used to transform INVαF´ cells that were plated on agar containing ampicillin (50 μg/mL) and tobramycin (10 μg/mL). The resulting colonies harbored plasmid p18MUR050 with an insert of ≈9 kb, which when sequenced showed *armA*, the structural gene for a 16S rRNA methylase (10). To confirm that *armA* was responsible for aminoglycoside resistance in this strain, 2 oligonucleotides, armF (5´-GGTGCGAAAACAGTCGTAGT-3´) and armR (5´-TCCTCAAAATATCCTCTATGT-3´), were used to amplify *armA*, which was purified, ligated into pUC18, and transformed into *E. coli* INVαF´ with selection on ampicillin and tobramycin. Transformant INVαF´ (p*armA*) was highly resistant to 4,6-disubstituted deoxystreptamines (Table), which demonstrated that *armA* was responsible for high-level aminoglycoside resistance in MUR050.

Until now, the *armA* gene has been found only on conjugative plasmids of the IncL/M incompatibility group and associated with the *bla*_CTX-M3_ gene coding for a broad-spectrum β-lactamase (11). Plasmid pMUR050, however, did not confer resistance to the cephalosporins and did not belong to the IncL/M family, as tested by PCR (data not shown), which indicated that *armA* was carried by a different replicon. Plasmid pMUR050 DNA was digested with *Sau3*A, and the resulting fragments were ligated with plasmid pBluescript KS+ DNA (Stratagene, La Jolla, CA, USA). The ligation mixture was transformed into *E. coli* INVαF´ and plated on agar containing ampicillin (50 μg/mL). The inserts of plasmids from 96 random clones were sequenced with standard oligonucleotides with an ABI Prism DNA Sequencer apparatus (Perkin-Elmer, Foster City, CA, USA). Approximately half of the sequence had a high degree of identity (80%–100%) with genes of conjugative plasmid R46, which belongs to the IncN incompatibility group (12). Among them, sequences involved in plasmid replication and mobilization, such as *repA* and *oriT*, were identified (data not shown), which confirmed that *armA* was carried by an R46-like replicon. This finding suggests that *armA* could be part of a mobile genetic element that has translocated between plasmids of diverse evolutionary origins. Upstream from *armA* an open reading frame (ORF) has 69% identity with that of a transposase from *Listonella anguillarum* (GenBank accession no. AAO92373); this ORF and *armA* are flanked by 16-bp directly repeated sequences with a single mismatch (5´-aggtttccactacagt-3´) (GenBank accession no. AY522431). However, since aminoglycosides are rarely used in porcine production in Spain (13), and no veterinary drugs containing amikacin are registered, the *sul1* and *armA* genes were conceivably co-selected by the use of sulfonamides. These antimicrobial agents are not among the 4 antimicrobial agents authorized in Europe for growth promotion (14) but are used orally to treat bacterial infections such as group E streptococcal pneumonia or atrophic rhinitis and diarrhea (2).

## Conclusions

These data support the notion that *armA* is disseminated both by conjugation and transposition, which makes further spread of *armA* likely. The complete sequence of plasmid pMUR050 is being determined and should help in elucidating the genetic basis for dissemination of *armA*.

The origin of the *armA* gene is still unknown. Aminoglycoside-producing environmental bacteria possess 16S rRNA methylases to protect themselves from the antimicrobial agent that they produce (15). In addition to ArmA (11), 2 other 16S rRNA methylases have been reported recently in human pathogens from Japan: RmtA in *Pseudomonas aeruginosa* (16) and RmtB in *Serratia marcescens* (17). One could speculate that actinomycetes have transferred the methylase genes to pathogenic bacteria in the environment that further spread on diverse replicons in various hosts. However, the degree of identity of *armA* with the structural genes for the 16S rRNA methylases in actinomycetes is <30%, which is not compatible with a recent transfer event. Alternatively, *armA* could have originated from a yet unknown aminoglycoside-producing actinomycetes (10).

In 2003, *armA* was identified as a new determinant that conferred high-level resistance to aminoglycosides in a French clinical isolate of *Klebsiella pneumoniae* (10) and was subsequently detected in human isolates of *Enterobacteriaceae* from various countries in Europe (11). The finding of *armA* in animal isolates shows that veterinary monitoring networks should include last-resort antimicrobial agents of high therapeutic value for humans, which could help in detecting early subtle shifts in antimicrobial resistance. In fact, *armA* was detected in an animal bacterium because amikacin was included in the standard antimicrobial panel in the Spanish Veterinary Antimicrobial Resistance Surveillance Network. Our finding of *armA* in a bacterium of animal origin highlights the importance of coordinated surveillance of human and animal isolates (18–20).
